# Diagnostic accuracy of the FRAIL scale plus functional measures for frailty screening: a cross-sectional study

**DOI:** 10.3399/BJGPO.2021.0220

**Published:** 2022-08-24

**Authors:** Ángel Rodríguez-Laso, Iñaki Martín-Lesende, Alan Sinclair, Sandrine Sourdet, Matteo Tosato, Leocadio Rodríguez-Mañas

**Affiliations:** 1 CIBERFES (Network-based Biomedical Research Consortium, area of Frailty and Healthy Ageing), Instituto de Salud Carlos III, Madrid, Spain; 2 Indautxu Primary Health Centre, Bilbao-Basurto Integrated Health Organisation, Basque Health Service (Osakidetza), Bilbao, Spain; 3 Foundation for Diabetes Research in Older People (fDROP), London, UK; 4 Medical Sciences, Nursing and Midwifery, Kings College, London, UK; 5 Department of Internal Medicine and Geriatrics, Toulouse University Hospital, Toulouse, France; 6 Fondazione Policlinico Universitario Agostino Gemelli IRCSS, Rome, Italy

**Keywords:** primary health care, frailty, frailty phenotype, mass screening

## Abstract

**Background:**

There is little knowledge of the diagnostic accuracy of screening programmes for frailty in primary care settings.

**Aim:**

To assess a two-step strategy consisting of the administration of the FRAIL scale to those who are non-dependent and aged ≥75 years, followed-up by measurement of the Short Physical Performance Battery (SPPB) or gait speed in those who are positive.

**Design & setting:**

Cross-sectional and longitudinal cohort study. Analysis of primary care data from the FRAILTOOLS project at five European cities.

**Method:**

All primary care patients consecutively attending were enrolled. They received the index tests, plus the Fried frailty phenotype (FP) and the frailty index to assess their frailty status. Mortality and worsening of dependency in basic and instrumental activities of daily living (BADL and IADL) over 1 year were ascertained.

**Results:**

Prevalence of frailty based on FP was 14.9% in the 362 participants. A FRAIL scale score ≥1 had a sensitivity of 83.3% (95% confidence interval [CI] = 73.1 to 93.6) to detect frailty. A positive result and an SPPB score <11 had a sensitivity of 72.2% (95% CI = 59.9 to 84.6); when combined with a gait speed <1.1 m/s, the sensitivity was 80.0% (95% CI = 68.5 to 91.5). Two-thirds of those screened as positive were not frail. In the best scenario, sensitivities of this last combination to detect IADL and BADL worsening were 69.4% (95% CI = 59.4 to 79.4) and 63.6% (95% CI = 53.4 to 73.9), respectively.

**Conclusion:**

Combining the FRAIL scale with other functional measures offers an acceptable screening approach for frailty. Accurate prediction of worsening dependency and death need to be confirmed through the piloting of a frailty screening programme.

## How this fits in

Busy primary care clinicians need a quick and easy-to-administer tool for frailty screening in older patients, whose positive results may ideally be confirmed with functional performance measures. There is no information on the diagnostic accuracy of such a strategy in primary care. The administration of the FRAIL scale plus the SPPB or the measurement of gait speed to non-dependent individuals aged ≥75 years had a good sensitivity to detect frailty, with an acceptable rate of false positives. Results suggest that it can also predict worsening of dependency in a 1-year period.

## Introduction

Frailty in older people is a progressive age-related decline in physiological systems resulting in decreased reserves of intrinsic capacity, extreme vulnerability to stressors, and increased risk of adverse health outcomes.^1^ Screening for this very common condition (35% of patients aged ≥70 years attend primary care in Europe)^2^ allows early detection and intervention before consequences occur, such as disability, which is more difficult to reverse. There is evidence on the validity, reliability, and feasibility of several tools to perform the screening and on the efficacy of interventions to reverse frailty, mainly multicomponent exercise.^3^

Several countries and regions have deployed screening programmes in primary care with different instruments that generate variable workloads for primary care teams.^3,4^ The usual limitation of attention time in these practices combined with possible limitations to face-to-face contact, like those brought about by the COVID-19 pandemic, suggest the need for a screening instrument that can be administered quickly and on the phone. The FRAIL scale^5^ meets these requirements and could be combined with performance tests, such as the SPPB^6^ or the measurement of gait speed, to confirm positive results. These are tools recommended by the ADVANTAGE Joint Action.^3^

To the authors' knowledge, there is no published evidence on the diagnostic accuracy of this strategy for frailty, worsening of dependency, or death screening. The objective of this study was to evaluate the sensitivity, specificity, and predictive values for this strategy. Different cut-offs for the three instruments were explored.

## Method

This article adheres to the STARD guidelines for reporting diagnostic accuracy studies.^7^

The design and rationale of the FRAILTOOLS project have been previously published in more detail.^8^ It was an observational, prospective, and longitudinal study planned to explore the diagnostic accuracy of several frailty instruments. It enrolled consecutively 1440 adults (aged ≥75 years) from primary care clinics, geriatric medicine services, and nursing homes from France (Toulouse), Italy (Rome), Poland (Cracow), Spain (Getafe), and the UK (Birmingham). Exclusion criteria were as follows: a Mini-Mental State Examination ≤20 points; a terminal illness (life expectancy ≤6 months); and a Barthel Index <90. Variables were collected at baseline in 2016 and at 6-, 12-, and 18-months‘ follow-up. This article is limited to the 381 primary care patients and their 1-year follow-up. The first five patients with no exclusion criteria attending primary care practices each morning were selected. These practices were mainly those that volunteered to participate among those that referred patients to the principal investigators’ affiliation hospitals.

Information on age, sex, multimorbidity,^9^ and several frailty instruments was collected at baseline. This article focuses on the following index tests and frailty measures.

The FP^10^ constitutes one of the reference standards because of its general acceptance as a measure of frailty.^3^ It consists of three self-reported components (exhaustion, physical activity, and weight loss), and two objective measures (grip strength and gait speed). Exhaustion was considered present when the responder answered at least 3–4 days during the past week to any of the following two questions from the Center for Epidemiological Studies Depression (CES-D) scale:^11^ ‘I felt that anything I did was a big effort’ and ‘I felt that I could not keep on doing things’. The physical activity item was considered present when males referred <2.5 hours walking per week (equivalent to <383 kcal) and women <2 hours (<270 kcal) usually. The weight loss item was considered positive if there was an unintentional loss of at least 4.5 kg in a year. Grip strength was measured as the best of three trials with a Jamar hydraulic dynamometer in the dominant hand. Gait speed was measured as the best of two trials at usual pace in a 4.5-metre distance from a standing position without using assisting devices. Both items were considered positive when the individual was in the worst quintile of strata of sex and body mass index for grip strength, and sex and height for gait speed.^12^ A patient was considered frail if ≥3 criteria were positive, even if the rest of the items were not measured. No imputation of missing items was performed.

The 35-item Frailty Index (FI-35) was the second reference standard because it belongs to the second recognised conceptualisation of frailty as an accumulation of deficits.^13^ It was calculated as the proportion of a list of health deficits (that is, symptoms, signs, chronic diseases, disability, and laboratory abnormalities) the patient suffered from,^14^ obtained from medical records, self-reported, or measured at the patient’s evaluation. The cut-off used to identify frailty was set to ≥0.25.^15^ According to the original protocol, the FI-35 allowed a missingness up to 20% of items to calculate the score and be able to classify the patient as frail.

The FRAIL scale, one of the index tests, comprises five self-reported items: Fatigue, Resistance, Ambulation, Illness, and Loss of weight.^5^ Although the recommended cut-off is ≥3, this article also explores lower ones. Any individual with any item lost was excluded from analyses.

Two performance measures, the two other index tests, were administered: the SPPB,^6^ which is a scale that ranges from 0–12 and combines three tests, gait speed, time to perform five chair stands, and balance assessment (in three positions: feet together, semi-tandem, and tandem); and gait speed (measured like the FP’s item).

For assessing predictive validity, the following frailty adverse outcomes were employed: 1) IADL and BADL dependency worsening at follow-up, which were defined as a loss of one point in the Lawton and Brody^16^ or a loss ≥5 points in the Barthel^17^ indexes, respectively; 2) death, which was ascertained through phone calls to arrange follow-up visits at 6 and 12 months and, when no answer was obtained, hospital registries (plus the death registry of the Ministry of Health in Spain).

All members of the research healthcare team (nurses and geriatricians) of all countries received the same training on the administration of the scales.^8^ All participants gave informed, written consent.

### Statistical analysis

Description of variables was carried out with medians and interquartile ranges (IQRs) or absolute and relative frequencies. Sensitivity, specificity, and percentage of positives who are not frail (false positives, understood as the complementary of the positive predictive value, not of the specificity) for frailty defined by the FP and the FI-35, and the three adverse outcomes were calculated for different thresholds of the FRAIL scale, and for different scores of the SPPB and increments of 0.1 m/s of gait speed in individuals with a FRAIL score ≥1.

The R package (version 4.0.2) was used for all analyses.

## Results

Out of the 381 primary care patients, 19 did not provide information on the index tests or frailty measures. There were more females, although the difference with participants was not statistically significant (*P* = 0.407). There were no differences in age (*P* = 0.49), Charlson Comorbidity Index (*P* = 0.323), nor Lawton and Brody scale (*P* = 0.437) and Barthel index (*P* = 0.326). The final sample size for the diagnostic accuracy of frailty was 362.

Characteristics of the sample for analyses are presented in Table 1. Median age was 79 years (IQR 77–82) and 58.8% were female. Three cities (Getafe, Toulouse, and Rome) contributed 79.2% of the sample. The median Charlson Comorbidity Index was high (median = 4; IQR 4–5). Dependency at baseline was infrequent, but around 17% became dependent after 1 year (loss to follow-up for this variable amounted to 24.0%). The prevalence of frailty was 14.9% (95% CI = 11.2 to 18.6) and 15.2% (95% CI = 11.5 to 18.9) according to the FP and the FI-35, respectively. Median SPPB score and gait speed were 10 and 1 m/s, respectively. Deaths were an extremely infrequent outcome, although missing information was the highest for this variable.

**Table 1.** Characteristics of the sample for the cross-sectional analysis (*n* = 362)

Category	Variable	*n* (%)^a^
Age, years, median (IQR)		79 (77–82)
Sex, female		213 (58.8)
City	Getafe (Spain)	116 (32.0)
	Toulouse (France)	93 (25.7)
	Rome (Italy)	78 (21.5)
	Cracow (Poland)	48 (13.3)
	Birmingham (UK)	27 (7.5)
Charlson Comorbidity Index score, median (IQR)		4 (4–5)
Barthel index score, median (IQR)		100 (95–100)
Lawton and Brody scale score, median (IQR)		8 (7–8)
Frail according to frailty phenotype^b^		54 (14.9)
Frail or prefrail according to frailty phenotype^b^	Total	276 (76.2)
	Missing	4 (1.1)
Number of items of the frailty phenotype^b^	0	82 (22.7)
	1	112 (30.9)
	2	104 (28.7)
	3	33 (9.1)
	4	17 (4.7)
	5	1 (0.3)
	Missing	13 (3.6)
Frailty index score, median (IQR)		0.16 (0.12–0.22)
Frail according to frailty index		55 (15.2)
Number of items of the FRAIL scale	0	209 (57.7)
	1	85 (23.5)
	2	38 (10.5)
	3	25 (6.9)
	4	4 (1.1)
	5	1 (0.3)
SPPB score, median (IQR)		10 (9–11)
Gait speed, m/s	Total, median (IQR)	1 (0.9–1.3)
	Missing	18 (5.0)
Worsening of dependence in basic activities of daily living	Total	46 (16.7)^c^
	Missing	87 (24.0)
Worsening of dependence in instrumental activities of daily living	Total	47 (17.1)^c^
	Missing	87 (24.0)
Deaths	Total	2 (0.8)^d^
	Missing	107 (29.6)

^a^Unless otherwise stated. ^b^These numbers do not match because of the possibility of assigning an individual with missing items to the frail or frail+ prefrail categories. ^c^ *N* = 275. ^d^ *N* = 255. IQR = interquartile range. SPPB = Short Physical Performance Battery.

Table 2 presents the prevalence and diagnostic accuracy of the FRAIL scale for three cut-off points. The traditional cut-off point (≥3) had a very low sensitivity for detecting frailty according to the FP (37.0%, 95% CI = 23.7 to 50.3). Decreasing the cut-off by one point rendered a higher sensitivity of 66.7% (95% CI = 53.7 to 79.7). Scoring any item of the FRAIL scale had a sensitivity of 83.3% (95% CI = 73.1 to 93.6). Indicators were slightly worse for frailty operationalised as the FI-35. Of the sample, 42.3% (95% CI = 37.2 to 47.4) scored at least one item of the FRAIL scale and therefore would be offered to be screened with the performance tests.

**Table 2.** Prevalence and diagnostic accuracy for frailty of different cut-offs of the FRAIL scale

FRAIL scale	Prevalence (95% CI)	Sensitivity (95% CI)		Specificity (95% CI)		% of positives who were not frail (95% CI)	
		Fried's frailty phenotype	Frailty index	Fried's frailty phenotype	Frailty index	Fried's frailty phenotype	Frailty index
≥3	8.3(5.4 to 11.1)	37.0(23.7 to 50.3)	23.6(12.0 to 35.2)	96.8(94.8 to 98.7)	94.5(91.9 to 97.0)	33.3(15.4 to 51.2)	56.7(37.8 to 75.5)
≥2	18.8(14.7 to 22.8)	66.7(53.7 to 79.7)	40.0(26.6 to 53.4)	89.6(86.2 to 93.0)	85.0(81.0 to 89.0)	47.1(34.9 to 59.2)	67.6(56.2 to 79.1)
≥1	42.3(37.2 to 47.4)	83.3(73.1 to 93.6)	74.5(62.7 to 86.4)	64.9(59.6 to 70.3)	63.5(58.1 to 68.9)	70.6(63.3 to 77.9)	73.2(66.1 to 80.3)

Table 3 presents the prevalence and diagnostic accuracy of the different scores of the SPPB among those with a score in the FRAIL scale ≥1. Percentages refer to the whole sample, not only those with a FRAIL score ≥1. A cut-off score of 10 yielded a sensitivity of 68.5% (95% CI = 55.7 to 81.3) to detect frailty operationalised with the FP. Rising the cut-off by one point increased the sensitivity to 72.2% (95% CI = 59.9 to 84.6) at the cost of increasing the proportion of false positives from 58.4% (95% CI = 48.0 to 68.9) to 66.7% (95% CI = 58.0 to 75.3). When the condition to be screened was defined as frailty according to the FI-35, the cut-off to obtain a similar sensitivity was <12. Using a cut-off of <11, 32.3% (95% CI = 27.5 to 37.2) of the total eligible population would be referred to a multidimensional evaluation.

**Table 3.** Prevalence and diagnostic accuracy for frailty of different SPPB scores in individuals with a FRAIL score ≥1

SPPB score	Prevalence (95% CI)	Sensitivity (95% CI)		Specificity (95% CI)		% of positives who were not frail (95% CI)	
		Fried's frailty phenotype	Frailty index	Fried's frailty phenotype	Frailty index	Fried's frailty phenotype	Frailty index
	0.6(0.0 to 1.3)	3.7(0.0 to 8.9)	3.6(0.0 to 8.7)	100.0	100.0	0.0	0.0
<2	0.8(0.0 to 1.8)	5.6(0.0 to 11.9)	3.6(0.0 to 8.7)	100.0	99.7(99.0 to 100.0)	0.0	33.3(0.0 to 100.0)
<3	1.1(0.0 to 2.2)	7.4(0.2 to 14.6)	5.5(0.0 to 11.7)	100.0	99.7(99.0 to 100.0)	0.0	25.0(0.0 to 100.0)
<4	1.9(0.5 to 3.4)	11.1(2.5 to 19.8)	7.3(0.2 to 14.4)	99.7(99.0 to 100.0)	99.0(97.9 to 100.0)	14.3(0.0 to 49.2)	42.9(0.0 to 92.3)
<5	3.0(1.3 to 4.8)	18.5(7.8 to 29.2)	10.9(2.4 to 19.4)	99.7(99.0 to 100.0)	98.4(96.9 to 99.8)	9.1(0.0 to 29.3)	45.5(10.4 to 80.5)
<6	5.5(3.2 to 7.9)	31.5(18.7 to 44.3)	21.8(10.6 to 33.1)	99.0(97.9 to 100.0)	97.4(95.6 to 99.2)	15.0(0.0 to 32.1)	40.0(16.5 to 63.5)
<7	7.2(4.5 to 9.9)	38.9(25.5 to 52.3)	23.6(12.0 to 35.2)	98.4(97 to 99.8)	95.8(93.5 to 98.0)	19.2(3.0 to 35.5)	50.0(29.4 to 70.6)
<8	10.2(7.1 to 13.4)	46.3(32.6 to 60.0)	30.9(18.3 to 43.5)	96.1(93.9 to 98.3)	93.5(90.7 to 96.3)	32.4(16.6 to 48.3)	54.1(37.2 to 70.9)
<9	15.7(12.0 to 19.5)	55.6(41.9 to 69.2)	40.0(26.6 to 53.4)	91.2(88.1 to 94.4)	88.6(85.0 to 92.2)	47.4(34.0 to 60.7)	61.4(48.4 to 74.4)
<10	24.6(20.1 to 29.0)	68.5(55.7 to 81.3)	56.4(42.8 to 69.9)	83.1(78.9 to 87.3)	81.1(76.7 to 85.5)	58.4(48.0 to 68.9)	65.2(55.1 to 75.3)
	32.3(27.5 to 37.2)	72.2(59.9 to 84.6)	67.3(54.5 to 80.1)	74.7(69.8 to 79.6)	73.9(69.0 to 78.9)	66.7(58.0 to 75.3)	68.4(59.8 to 76.9)
<12	38.4(33.4 to 43.4)	75.9(64.1 to 87.7)	72.7(60.6 to 84.9)	68.2(63.0 to 73.4)	67.8(62.5 to 73.0)	70.5(62.8 to 78.2)	71.2(63.6 to 78.8)

Percentages refer to the total sample (*n* = 362), not only to individuals with a FRAIL score ≥1. SPPB = Short Physical Performance Battery.

Table 4 presents the same structure as Table 3 but refers to different gait speeds. Sample size was smaller (*N* = 342) because in some cases gait speed was ascertained for the SPPB scoring but was not recorded in m/s. A FRAIL score of ≥1 plus a gait speed <0.8 m/s showed a sensitivity for frailty of only 52.0% (95% CI = 37.7 to 66.3) for the FP and 34.7% (95% CI = 20.9 to 48.5) for the FI-35. Sensitivity reached 74.0% at a cut-off <1 m/s and got higher at the expense of a little increase of false positives at <1.1 m/s (sensitivity 80.0%, 95% CI = 68.5 to 91.5; false positives 67.7%, 95% CI = 59.4 to 76.1). Of the sample, 36.3% (95% CI = 31.1 to 41.4) walked at this speed. A cut-off of <1.2 m/s increased sensitivity and false positives by 2%. For the FI-35, starting from <1 m/s increments in sensitivity and false positives were parallel.

**Table 4.** Prevalence and diagnostic accuracy for frailty of different gait speeds in individuals with a FRAIL score ≥1

Gait speed, m/s	Prevalence (95% CI)	Sensitivity (95% CI)		Specificity (95% CI)		% of positives who were not frail (95% CI)	
		Fried's frailty phenotype	Frailty index	Fried's frailty phenotype	Frailty index	Fried's frailty phenotype	Frailty index
<0.6	6.7(4.1 to 9.4)	36.0(22.2 to 49.8)	24.5(12.0 to 37.0)	98.3(96.8 to 99.8)	96.2(94.1 to 98.4)	21.7(3.5 to 40.0)	47.8(25.7 to 69.9)
<0.7	9.6(6.5 to 12.8)	40.0(25.9 to 54.1)	24.5(12.0 to 37.0)	95.5(93.2 to 97.9)	92.8(89.9 to 95.8)	39.4(21.8 to 57.0)	63.6(46.3 to 81.0)
<0.8	16.4(12.4 to 20.3)	52.0(37.7 to 66.3)	34.7(20.9 to 48.5)	89.7(86.2 to 93.2)	86.7(82.8 to 90.6)	53.6(40.1 to 67.0)	69.6(57.2 to 82.1)
<0.9	23.7(19.2 to 28.2)	62.0(48.1 to 75.9)	57.1(42.8 to 71.5)	82.9(78.5 to 87.2)	81.9(77.5 to 86.3)	61.7(50.9 to 72.5)	65.4(54.9 to 76.0)
<1	33.0(28.0 to 38.1)	74.0(61.4 to 86.6)	69.4(56.0 to 82.8)	74.0(68.9 to 79.0)	73.0(67.9 to 78.1)	67.3(58.5 to 76.0)	69.9(61.3 to 78.5)
<1.1	36.3(31.1 to 41.4)	80.0(68.5 to 91.5)	71.4(58.3 to 84.5)	71.2(66.0 to 76.5)	69.6(64.3 to 74.9)	67.7(59.4 to 76.1)	71.8(63.7 to 79.8)
<1.2	39.5(34.3 to 44.7)	82.0(71.0 to 93.0)	73.5(60.7 to 86.3)	67.8(62.4 to 73.2)	66.2(60.8 to 71.7)	69.6(61.8 to 77.5)	73.3(65.8 to 80.9)
<1.3	41.5(36.3 to 46.8)	82.0(71.0 to 93.0)	75.5(63.0 to 88.0)	65.4(59.9 to 70.9)	64.2(58.6 to 69.7)	71.1(63.6 to 78.7)	73.9(66.6 to 81.3)
<1.4	42.1(36.8 to 47.4)	82.0(71.0 to 93.0)	75.5(63.0 to 88.0)	64.7(59.2 to 70.2)	63.5(57.9 to 69.0)	71.5(64.1 to 79.0)	74.3(67.1 to 81.5)
<1.5	42.4(37.1 to 47.7)	82.0(71.0 to 93.0)	75.5(63.0 to 88.0)	64.4(58.9 to 69.9)	63.1(57.6 to 68.7)	71.7(64.3 to 79.1)	74.5(67.3 to 81.7)

Percentages refer to the total sample (*n* = 342), not only to individuals with a FRAIL score ≥1.

Out of the 362 individuals in the cross-sectional analyses, 87 could not be assessed for their final dependency status, rendering a sample size of 275. Compared with those assessed, those lost were 1-year older (*P* = 0.037). Their Charlson Comorbidity Index (*P* = 0.124), Lawton and Brody scale (*P* = 0.319), and Barthel (*P* = 0.735) baseline indexes were similar, but frailty according to the FP was more common (21% versus 13%, *P* = 0.049) and the average number of positive items of FRAIL scale mildly higher (0.23; *P* = 0.059).

A score of the FRAIL scale ≥1 had a sensitivity of 52.2% (95% CI = 37.2 to 67.2) and 46.8% (95% CI = 32.0 to 61.6) to detect a 1-year worsening of BADLs and IADLs, respectively. Combining it with the SPPB or gait speed, sensitivities would get even lower.

Sensitivity analysis was performed by handling missing data in the worsening dependency variables by the best-case imputation, where missing cases were considered to have worsened their dependency if their FRAIL scale score was >0 at baseline, and not considered to have worsened their dependence if their FRAIL scale score was 0. Under this assumption, FRAIL score ≥1 sensitivities increased to 76.1% (95% CI = 67.2 to 85.0) for BADL and 73.1% (95% CI = 63.9 to 82.3) for IADL. A FRAIL score ≥1 plus SPPB <11 had a sensitivity of 60.9% (95% CI = 50.7 to 71.0) and false positives of 52.1% (95% CI = 43.0 to 61.3) to predict worsening of BADL, and of 59.1% (95% CI = 49.0 to 69.3) and 53.0% (95% CI = 43.8 to 62.2) to predict worsening of IADL. A FRAIL score ≥1 plus gait speed <1.1 m/s had a sensitivity of 63.6% (95% CI = 53.4 to 73.9) and false positives of 54.8% (95% CI = 46.0 to 63.7) to predict worsening of BADL, and of 69.4% (95% CI = 59.4 to 79.4) and 52.4% (95% CI = 43.5 to 61.3) to predict worsening of IADL. Supplementary Tables S1 and S2 present results for other cut-offs.

Death could not be ascertained in 107 people of the cross-sectional sample, which left a sample size for the diagnostic accuracy of mortality of 255 individuals. Two deaths occurred during the follow-up, both with a FRAIL score of 1, SPPB scores of 6 and 9, and gait speeds of 0.79 m/s and 0.67 m/s, respectively. That means that the sensitivity for death of a FRAIL score ≥1 and SPPB <11 or any of the proposed cut-offs for gait speed was 100%. Nevertheless, 97.2% (95% CI = 93.3 to 100) of those considered positive under the former criteria did not die.

## Discussion

### Summary

This article shows that a strategy, which screens all non-dependent adults aged ≥75 years in primary care with the FRAIL scale and follows-up positive results with the SPPB or measurement of gait speed, has a reasonable diagnostic accuracy for frailty detection when the following thresholds are applied: a positive answer to any of the items of the FRAIL scale (instead of the recommended cut-off of ≥3 items), plus an SPPB score <11 or gait speed <1.1 m/s (instead of the usual threshold of 0.8 m/s). The results suggest that this strategy may also predict those who worsen their dependency level in 1-year's time, although cautiousness in the interpretation is warranted because of losses to follow-up.

### Strengths and limitations

The article has the strength of presenting the diagnostic accuracy results of a previously untested frailty screening strategy carried out in a multi-country sample of primary care patients. Its main limitation is loss to follow-up, which did not allow the authors to obtain conclusive results on prediction of dependency worsening because of the discrepancies in results when non-imputing and imputing by the best-case approach. It is known that lost-to-follow-up individuals were older and marginally frailer, which limits the capacity of generalising the results to all eligible users. The authors believe most of these individuals probably dropped out from the study because of tiredness and loss of motivation owing to the long administration time to perform the full frailty assessment in FRAILTOOLS with seven instruments. Another limitation is a short follow-up to detect deaths, but extending analyses to 18 months, as stablished in the FRAILTOOLS protocol, would have increased missingness.

### Comparison with existing literature

Our results of the sensitivity of the FRAIL scale are comparable to those published by Ambagtsheer *et al* ^18^ from a study in primary care patients aged ≥75 years in southern Australia, where they reported a sensitivity of 30% (95% CI = 16.6 to 46.5) and 19.8% (95% CI = 12.9 to 28.5) for the FP and the frailty index, respectively, with a cut-off of ≥3. They are lower than those reported in eastern China, where sensitivity results for the FP in community dwellers aged ≥60 years were 52.2% for a score ≥3, 87% for a score ≥2, and 97.8% for a score ≥1.^19^ Similar results to Dong *et al^19^* were reported by Thompson *et al* ^20^ for the FP in community dwellers from the northwest of Australia aged ≥65 years. In relation to prediction of disability worsening, Si *et al* ^21^ found a sensitivity of a score of the FRAIL scale ≥3 at 1-year follow-up of 11.7% for BADL and 9.9% for IADL in community dwellers aged ≥60 years from a Chinese city. They did not offer results for lower cut-off points.

Although the SPPB has been used for screening in primary care,^22^ data have not been recorded about its ability to detect frailty nor adverse outcomes in this level of attention. Ambagtsheer *et al* ^18^ also studied the diagnostic accuracy of gait speed ≤0.8 m/s (at four-metre distance). Sensitivity against the FP and the frailty index was 70.0% (95% CI = 53.5 to 83.4) and 47.8% (95% CI = 38.2 to 57.4), respectively, and specificity 77.1% (95% CI = 70.5 to 82.9) and 84.6% (95% CI = 76.8 to 90.6), respectively. Our sensitivities are lower and specificities higher because we added the requirement of having a positive answer in any of the items of the FRAIL scale.

### Implications for research and practice

**Figure 1.**
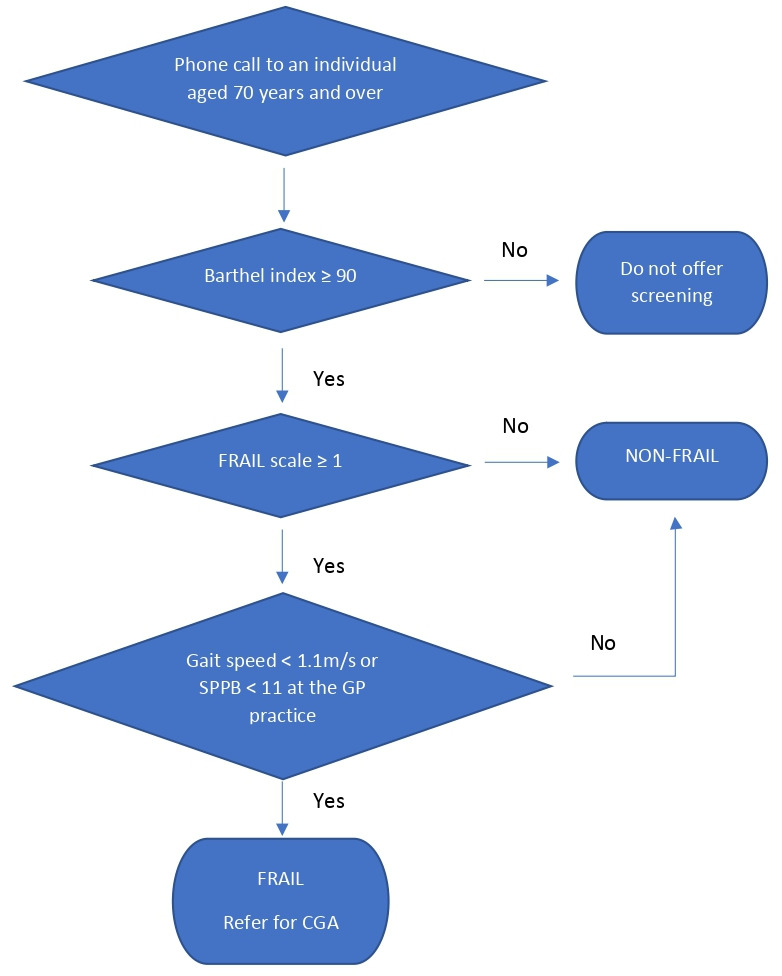
Recommended frailty screening algorithm in primary care. CGA = comprehensive geriatric assessment. SPPB = Short Physical Performance Battery.

The use of the FRAIL scale as a screening tool for frailty has many advantages for busy primary care clinics; for example, it has a short administration time (less than a minute and a half in most cases),^23^ requires little training or instruction for the assessor, and can be delivered over the phone. The authors recommend using it in the context of the algorithm presented in Figure 1. Although the results were limited to individuals aged ≥75 years, existing recommendations have been adhered to^3,4^ and all individuals aged ≥70 years have been considered. Any positive answer to the items of the FRAIL scale over the phone in a non-dependent patient should elicit an in-person consultation where either the SPPB or gait speed would be measured. The authors predict that less than half of the screened population will require to be referred to functional assessment, which would certainly reduce primary care teams’ workload compared with the assessment of all individuals with functional measures (as recommended in other screening programmes).^24^ Positive results in functional assessments should be confirmed through a comprehensive geriatric assessment (CGA) carried out in the primary or secondary level of attention, something that will be required by around one-third of the eligible population. The CGA should encompass the prescription of a multicomponent exercise intervention in confirmed cases.^3^ Cut-offs that the authors consider acceptable considering the sensitivity, proportion of false positives, and workload that they would produce are suggested here. These decisions should be tempered by the resources available to carry out functional measurements and CGAs.

To increase certainty on the ability of this strategy to predict death and dependency worsening, a pilot programme with usual primary care users followed for a longer period is warranted.

NHS England opted for detecting frailty in primary care following the accumulation of deficits paradigm using electronic medical records.^25^ This would be equivalent in the present study to just administering the FI-35 to the whole sample. This is not a screening strategy, but a diagnostic one, because all cases according to this definition of frailty would be detected. Curiously enough, NHS England states nevertheless that *‘confirmation of frailty in an individual should be undertaken using a validated tool such as:* [the] *Gait Speed Test*’.^26^ They also state, in another document:^25^ *‘... a clinician from the primary care team should verify the frailty diagnosis by direct assessment using the Clinical Frailty Scale (CFS) or similar validated tool’.* Both instruments are considered screening tests for frailty, not diagnostic tools.^3^ In any case, the FP and index approaches have different purposes and are to be considered complementary in the evaluation of the older person.^27^ One of their main differences is that the frailty index includes diseases, disability, and dependency items, while the FP was conceived as a measure of a condition that usually precedes disability, and because of that it is based on assessing performance-based tasks, which are different from disability. It has been shown that the algorithm is more sensitive to detect frailty according to the FP rather than the frailty index, and therefore more suitable to identify patients at risk of developing disability.
